# Combining Sodium Butyrate With Cisplatin Increases the Apoptosis of Gastric Cancer *In Vivo* and *In Vitro* via the Mitochondrial Apoptosis Pathway

**DOI:** 10.3389/fphar.2021.708093

**Published:** 2021-08-27

**Authors:** Yangbo Li, Pengzhan He, Yinghui Liu, Mingming Qi, Weiguo Dong

**Affiliations:** ^1^ Department of Gastroenterology, Renmin Hospital of Wuhan University, Wuhan, China; ^2^ Central Laboratory, Renmin Hospital of Wuhan University, Wuhan, China; ^3^ Key Laboratory of Hubei Province for Digestive System Disease, Wuhan, China

**Keywords:** sodium butyrate, cisplatin, gastric cancer, apoptosis, mitochondrial pathway

## Abstract

**Introduction:** The gastrointestinal malignancy, gastric cancer (GC), has a high incidence worldwide. Cisplatin is a traditional chemotherapeutic drug that is generally applied to treat cancer; however, drug tolerance affects its efficacy. Sodium butyrate is an intestinal flora derivative that has general anti-cancer effects in vitro and in vivo via pro-apoptosis effects and can improve prognosis in combination with traditional chemotherapy drugs. The present study aimed to assess the effect of sodium butyrate combined with cisplatin on GC.

**Methods:** A Cell Counting Kit-8 assay was used to assess the viability of GC cells in vitro. Hoechst 33,258 staining and Annexin V-Phycoerythrin/7-Aminoactinomycin D were used to qualitatively and quantitatively detect apoptosis in GC cells. Intracellular reactive oxygen species (ROS) measurement and a mitochondrial membrane potential (MMP) assay kit were used to qualitatively and quantitatively reflect the function of mitochondria in GC cells. Western blotting was used to verify the above experimental results. A nude mouse xenograft tumor model was used to evaluate the anti-tumor efficacity of sodium and cisplatin butyrate in vivo.

**Results:** Cisplatin combined with sodium butyrate increased the apoptosis of GC cells. In the nude mouse xenograft tumor model, sodium butyrate in combination with cisplatin markedly inhibited the growth of the tumor more effectively than either single agent. The combination of sodium butyrate and cisplatin increased the intracellular ROS, decreased the MMP, and suppressed the invasion and migration abilities of GC cells. Western blotting verified that the combination of sodium butyrate and cisplatin remarkably enhanced the levels of mitochondrial apoptosis-related pathway proteins.

**Conclusion:** Sodium butyrate, a histone acetylation inhibitor produced by intestinal flora fermentation, combined with cisplatin enhanced the apoptosis of GC cells through the mitochondrial apoptosis-related pathway, which might be considered as a therapeutic option for GC.

## Introduction

Gastric cancer (GC) is one of the most common gastrointestinal malignancies and ranks fifth in the incidence of malignancies worldwide. In 2020, the number of deaths caused by gastric cancer was estimated to exceed 769,000, and its mortality rate ranks only behind lung cancer and liver cancer according to the latest data from GLOBOCAN 2020 (Sung et al., 2021). Although a recent study showed that GC incidence and mortality rates have continued to decline globally (Luo et al., 2017), stomach cancer remains a heavy health burden in China. In 2018, 10.6% of all cases of GC occurred in China, and the 5-year survival rate was quite low, at less than 35% from 2013 to 2015 (Zeng et al., 2018). Currently, the major treatment modalities for GC are combination therapy, radiotherapy, and surgery; however, the fact that 70% of patients with GC are diagnosed as suffering from terminal cancer greatly limits the effectiveness of treatment (Song et al., 2017). Drug resistance to cisplatin and 5-fluorouracil, for example, which are common traditional therapies, has resulted in progressively poor curation outcomes. Therefore, it is necessary to find new natural anticancer drugs with low toxicity and high efficiency to construct new chemotherapy regimen combinations to avoid worsening drug resistance.

Sodium butyrate (NaB), a derivative of butyric acid, is a metabolite produced by the breakdown of fiber in food residues by intestinal microorganisms (Sanna et al., 2019). Sodium butyrate not only regulates intestinal function, provides energy to intestinal epithelial cells, and regulates cell flora but also acts as an anti-inflammatory factor to maintain intestinal homeostasis (Bardhan et al., 2015). In addition, sodium butyrate is a natural histone deacetylase inhibitor (HDACi). HDACis are a new class of oncology chemotherapeutic drugs that have shown enhanced efficacy and reduced toxicity in combination with classical therapies (Guerriero et al., 2017). Scholars have shown experimentally that sodium butyrate can inhibit proliferation and promote apoptosis *in vivo* and *in vitro* of a variety of tumor cells, such as bladder cancer (Wang F. et al., 2020), lung cancer (Xiao et al., 2020), and colorectal cancer (Wang W. et al., 2020; Xi et al., 2021). Sodium butyrate inhibits tumor growth through multiple mechanisms, particularly the mitochondrial apoptosis pathway (Salimi et al., 2017; Qin et al., 2020). Encouragingly, sodium butyrate made tumor cells more sensitive to the anticancer drug, docetaxel (Chen et al., 2020). Previous studies showed that sodium butyrate decreased the focal adhesion kinase (FAK) expression by increasing the death associated protein kinase (DAPK) levels in GC cells (Shin et al., 2012), potentially inducing the cell-cycle inhibitors, cyclin dependent kinase inhibitor 1A (CDKN1A, also known as p21Waf1/Cip1), and cyclin dependent kinase inhibitor 1B (CDKN1B, also known as p27Kip1), as well as the pro-apoptotic genes, *BAX* (encoding BCL2 associated X, apoptosis regulator), *BAK* (encoding BCL2 antagonist/killer), and *BIK* (encoding BCL2 interacting killer) in GC cells, which contributed to apoptosis (Litvak et al., 2000). However, the anti-tumor effect of sodium butyrate in combination with cisplatin in GC and its underlying mechanism remain unknown.

In the present study, GC cells were treated with sodium butyrate and cisplatin separately and in combination. The results showed that sodium butyrate inhibited the proliferation and promoted the apoptosis in GC cells by activating the mitochondrial apoptosis pathway.

## Materials and Methods

### Cell Culture

The China Center for Type Culture Collection (CCTCC) provided the human GC cell lines (HGC-27, SGC-7901, and MGC-803) and the normal cell line (GES-1). The cells were cultured in Dulbecco’s modified Eagle’s medium (DMEM)/F-12 medium (1:1) (HyClone, Logan, UT, United States) and Roswell Park Memorial Institute (RPMI) 1,640 medium (Gibco, Grand Island, NY, United States) with 10% fetal bovine serum (FBS) (Gibco) and a 1% solution of antibiotics (penicillin at 100 U/ml and streptomycin at 100 g/ml) (Beyotime, Jiangsu, China) at 37°C and 5% CO_2_ in a humidified incubator.

### Reagents and Antibodies

Sodium butyrate (>99% purity) and cisplatin were obtained from Sigma-Aldrich (St. Louis, MO, United States). Sodium butyrate was dissolved in ultrapure water to prepare a 900 mM stock solution and cisplatin was dissolved in absolute dimethyl sulfoxide (DMSO) to prepare a 4 mg/ml (4 mg cisplatin +1 ml DMSO) stock solution, both of which were stored at −20°C.

Rabbit monoclonal antibodies against B-cell lymphoma 2 (BCL-2), BCL2 associated X (BAX), Cytochrome C (CytC), apoptotic protease activating factor-1 (Apaf-1), apoptosis inducing factor (AIF), proliferating cell nuclear antigen (PCNA), cleaved caspase-3, cleaved caspase-9, matrix metalloproteinase (MMP)-2, MMP-9, survivin, and glyceraldehyde-3-phosphate dehydrogenase (GAPDH) were obtained from Cell Signaling Technology (Danvers, MA, United States). The antibodies are diluted to a working concentration at a ratio of 1:1,000 and stored at 4°C. The secondary antibodies were used at a working concentration of 1:10,000 and were obtained from LI-COR (Lincoln, NE, United States).

### Cell Proliferation Assay

The cell proliferation and viability were assessed quantitatively using a Cell Counting Kit-8 (CCK-8, Beyotime, Shanghai, China) *in vitro*. SGC-7901, HGC-27, MGC-803, and GES-1 cells (normal gastric mucosa epithelial cells) were sown in 96-well plates (at 5 × 10^3^ cells/well) and cultured for 24 h. Then, the GC cells were treated initially with different concentrations of sodium butyrate (0, 1, 2, 4, 8, 16, 32, and 64 mM), different concentrations of cisplatin (0, 1, 2, 4, 8, 16, 32, and 64 µg/ml), or a combination of cisplatin (0, 1, 2, 4, 8, 16, 32, and 64 µg/ml) and sodium butyrate (0.5 mM or 0, 1, 2, 4, 8, 16, 32, and 64 mM) for additional 24 h. The next day, we aspirated the supernatant liquid of each well, added 10 µL of CCK-8 solution, and continued the incubation for 2 h. Meanwhile, the control cells were incubated in DMEM/F-12 medium containing 10% CCK-8. Finally, a microplate reader (Victor3 1,420 Multilabel Counter, Perkin Elmer, Waltham, MA, United States) was used to measure the absorbance of each sample at 450 nm. GraphPad Prism software (GraphPad Inc., La Jolla, CA, United States) was used to calculate the half maximal inhibitory concentration (IC50) and CompuSyn software (CompuSyn Inc., Paramus, NJ, United States), which is based on the Chou-Talalay method, was used to obtain the combination index (CI) and to construct fraction affected (Fa)-CI plots. CI < 1, CI = 1, and CI > 1 represent synergistic, additive, and antagonistic effects, respectively, and the experiment was repeated three times in parallel.

### Transwell Invasion Assay

The GC cells were digested using trypsin and 100 µL of the cell suspension (5 × 10^4^ cells) was seeded on the upper chamber of a Transwell insert (Corning Costar Corp, Corning, NY, United States) with 8 µm pores that were precoated with Matrigel (BD Biosciences, San Jose, CA, United States). The next step was to add 600 µL of DMEM/F-12 medium with 15% FBS into the lower chamber and then the insert was incubated overnight in the incubator. The HGC-27 cells were treated with DDP (4 µg/ml), NaB (10 mM), or DDP (4 µg/ml) plus NaB (10 mM). Meanwhile, the SGC-7901 cells were treated with DDP (4 µg/ml), NaB (5 mM), or DDP (4 µg/ml) plus NaB (5 mM). After 24 h of incubation, the Transwell insert was put into a 24-well plate with 600 µL 4% paraformaldehyde to fix the cells for 20 min; then 0.2% crystal violet was used to stain the cells. After washing three times with phosphate-buffered saline (PBS), the Transwell inserts were observed in five random fields for each sample to count the invasive cells under an inverted microscope (BX51; Olympus Corporation, Tokyo, Japan).

### Wound-Healing Assay

The cells were seeded into a 6-well plate chamber (5 × 10^5^ cells/well) with fresh medium and incubated for 24 h. The next day, when the cells were spread evenly over the well, a 200 µL pipette tip was used to make a scratch in the cell monolayer. PBS was used to clean floating debris and then the wound was photographed immediately (0 h). The HGC-27 cells on the plates were then cultured in DMEM/F-12 medium with 10% FBS together with DDP (4 µg/ml), NaB (10 mM), or DDP (4 µg/ml) plus NaB (10 mM). Meanwhile, the SGC-7901 cells on the plates were cultured in DMEM/F-12 medium with 10% FBS together with DDP (4 µg/ml), NaB (5 mM), or DDP (4 µg/ml) plus NaB (5 mM). The wounds were photographed at 48 h and the area of wound healing was measured.

### Hoechst 33,258 Detection of the Apoptotic Cells

We examined cell apoptosis using a Hoechst 33,258 Staining Kit (Beyotime). HGC-27 cells and SGC-7901 cells in the exponential growth phase were seeded into a 6-well plate (1 × 10^5^ cells/well) and incubated for 24 h. The GC cells were treated with NaB and cisplatin as mentioned in "Transwell Invasion Assay" section.4 and then stained with Hoechst 33,258 according to the manufacturer’s instructions. The morphological features of apoptosis, such as chromatin condensation and nuclear fragmentation, were observed using fluorescence microscopy (BX51, Olympus).

### Apoptosis Analysis Using Annexin V-PE/7AAD Double Staining

An Annexin V-Phycoerythrin (PE)/7-Aminoactinomycin D (7AAD) kit (MultiSciences, Hangzhou, China) and flow cytometry (FACSCalibur, Becton Dickinson, Franklin Lakes, NJ, United States) were used to quantify the percentage of apoptotic cells. The cells were seeded into a 6-well plate and incubated for 24 h with different treatments and with or without pretreatment with N-acetylcysteine (NAC) or buthionine sulphoximine (BSO) for 2 h. According to the instructions, the adherent cells were collected and washed with PBS and finally co-stained with 5 µL Annexin V-PE and 5 µL 7AAD in binding buffer before the flow cytometry analysis. The cells were divided into four cell populations based on their various fluorescence characteristics: necrotic cells (Annexin V-PE− and 7AAD+), live cells (Annexin V-PE− and 7AAD−), late apoptotic cells (Annexin V-PE+ and 7AAD+), and early apoptotic cells (Annexin V-PE+ and 7AAD−).

### Measuring the ROS Levels

A ROS Assay Kit (Beyotime) was used to measure the ROS levels using 2ʹ,7ʹ-dichloro-fluorescein diacetate (DCFH-DA). The cells were seeded into a 24-well plate (1 × 10^5^ cells/well) and then exposed to DDP or NaB at different concentrations, as mentioned in "Transwell Invasion Assay" section, for 24 h. The next day, the cells were incubated with 10 µmol/L DCFH-DA for 20 min at 37°C in the dark. After being washed with PBS three times, coverslips were attached to the glass slides, and the cells were observed under an upright fluorescence microscope (Olympus).

### Measuring the Mitochondrial Membrane Potential

The changes to the mitochondrial membrane potential (△Ψm) were assessed using a Mitochondrial Membrane Potential Assay Kit with JC-1 (Beyotime). After the cells were treated, as mentioned in "Transwell Invasion Assay" section, for 24 h, the supernatant was removed and the cells were treated with JC-1 dye for 1 h. Finally, the cells were washed with buffer solution twice before observation under a laser confocal fluorescence microscope (Olympus) or were detected and analyzed using a flow cytometer.

### Western Blotting Analysis

After treatment with DDP or NaB, as mentioned in "Transwell Invasion Assay" section, for 24 h, the cell proteins were extracted in RIPA buffer supplemented with phenylmethylsulfonyl fluoride (PMSF) and protease inhibitors (Beyotime). A bicinchoninic acid (BCA) Protein Assay Kit (Beyotime) was then used to determine the protein concentrations according to the manufacturer’s instructions. The cellular proteins were separated by 10% SDS-PAGE and transferred to polyvinylidene difluoride (PVDF) membranes (Millipore, Billerica, MA, United States). Immediately, the membranes were blocked with 5% non-fat dry milk in Tris-buffered saline-Tween 20 (TBST) for 1 h. Then, the membranes were incubated with primary rabbit antibodies at 4°C overnight. After washing with TBST four times (5 min each time), the membranes were further incubated with secondary antibodies for 2 h at room temperature. The membranes were then washed with TBST four times (5 min each time). The Odyssey infrared imaging system (LI-COR) was used to scan the membranes to determine the immunoreactive protein bands. GAPDH was used as a protein loading control.

### Xenograft Tumor Models *In Vivo* and the TUNEL Assay

The Ethics Committee of Renmin Hospital of Wuhan University approved the study protocol and all the animal research procedures were performed according to the institutional ethical standards and/or those of the national research committee and according to the 1964 Helsinki Declaration and its later amendments or comparable ethical standards. The collected SGC-7901 cells were washed in serum-free DMEM, suspended in 100 µL of PBS, and implanted subcutaneously into the dorsal area of male BALB/c nude mice (5 weeks old), purchased from Beijing Life River Experimental Animal Technology Co. Ltd. (Beijing, China). When the tumor volume was approximately 100–150 mm^3^, the nude mice were randomly divided into four groups (n = 6 per group), which were treated via intraperitoneal injection with normal saline, DDP (4 mg/kg), NaB (200 mg/kg), or DDP (4 mg/kg) plus NaB (200 mg/kg) every 2 days. The mouse weight and the tumor volume were measured after each treatment time. The tumor volume (TV) was calculated using the following formula: TV (mm^3^) = d^2^ × D/2, where d and D represent the shortest and longest diameters, respectively. After 15 days, the mice were sacrificed humanely and their tumors were harvested and weighed. A terminal deoxynucleotidyl transferase dUTP nick end labeling (TUNEL) assay was performed using an apoptosis detection kit (Roche Applied Science, Basel, Switzerland) to detect the apoptotic cells in tumor tissue sections. To measure liver and renal function, we collected mouse blood to detect the activation of alanine aminotransferase (ALT) and aspartate aminotransferase (AST), and blood urea nitrogen (BUN) and serum creatinine (Cr) levels.

### Statistical Analysis

SPSS 21.0 software (IBM Corp., Armonk, NY, United States) was used for statistical analysis. The data were expressed as the mean ± SD, and ANOVA was used for comparison between groups. *p* < 0.05 was considered statistically significant.

## Results

### Sodium Butyrate Combined With Cisplatin Inhibited the Growth of GC Cells

**FIGURE 1:**
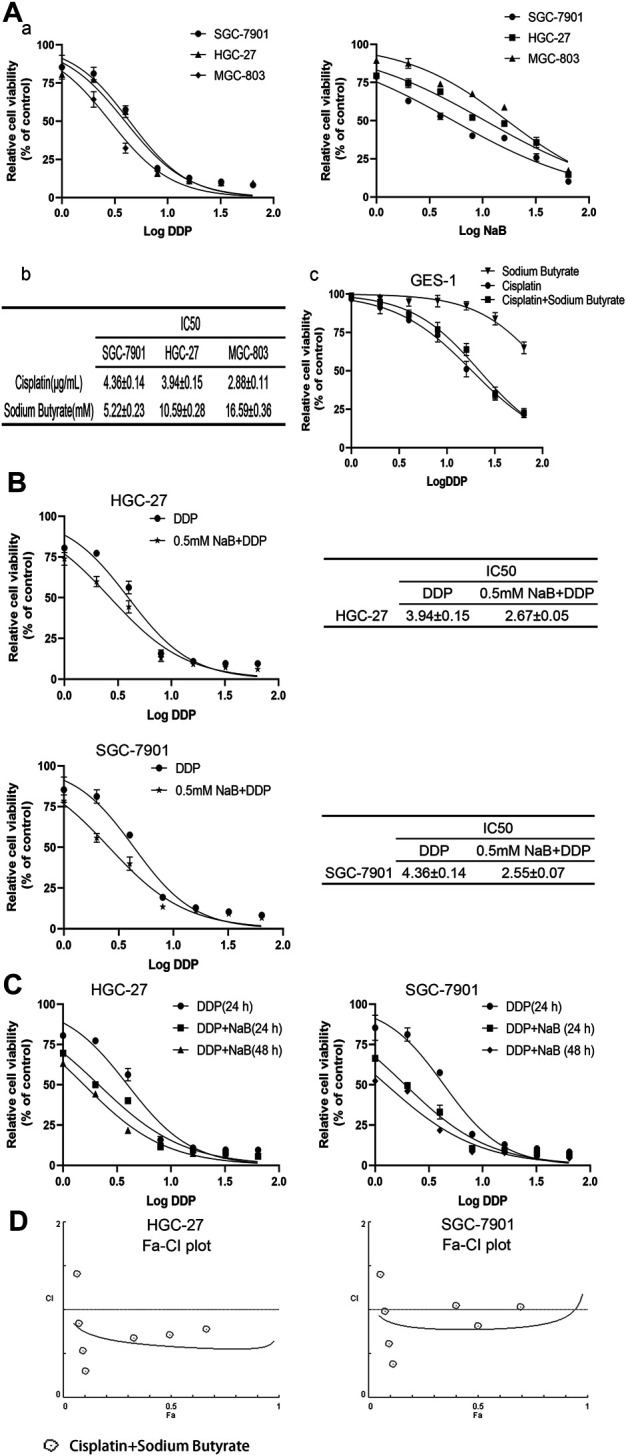
Evaluation of GC cells’ growth inhibition induced by cisplatin and/or sodium butyrate by CCK-8 kit. **(A)** (a) GC cells (HGC-27, SGC-7901 and MGC-803) were treated with cisplatin (0, 1, 2, 4, 8, 16, 32, and 64 µg/ml) or sodium butyrate (0, 1, 2, 4, 8, 16, 32, and 64 mM) for 24 h. (b) Cell Counting Kit-8 assays showed that HGC-27 cells and SGC-7901 cells were more sensitive to sodium butyrate. (c) GES-1 cells were treated with the different concentrations of cisplatin or sodium butyrate as described above and a combination of cisplatin and sodium butyrate for 24 h. **(B)** HGC-27 cells and SGC-7901 cells were treated with cisplatin and a combination of cisplatin and sodium butyrate (0.5 mM) for 24 h, respectively. **(C)** HGC-27 cells and SGC-7901 cells were treated with cisplatin and a combination of cisplatin and sodium butyrate for 24 h or 48 h. **(D)** CompuSyn software was used to define the type of drug-combination effect. All the above data are shown as the mean ± SD from an average of three experiments.

The GC cells and GES-1 cells were exposed to different concentrations of sodium butyrate or cisplatin or both for 24 h. All 3 GC cell lines had different sensitivities to cisplatin and sodium butyrate. There was no significant effect on the viability of 90% of GC cells when the concentration of sodium butyrate was 0.5 mM, and the measured IC50 values are shown in Figures 1Aa,b. There was no significant cytotoxicity to GES-1 when the concentration of sodium butyrate was below 32 mM (Figure 1Ac). The combination of sodium butyrate with cisplatin decreased the IC50 value of cisplatin and attenuated the cytotoxic effect of cisplatin on the normal cells. Accordingly, we selected SGC-7901 cells and HGC-27 cells, which showed better sensitivities to sodium butyrate to perform the subsequent experiments. The IC50 values of DDP and sodium butyrate in the SGC-7901 cells at 24 h were about 4 µg/ml and 5 mM, respectively. Meanwhile, the IC50 values of DDP and sodium butyrate in the HGC-27 cells at 24 h were about 4 µg/ml and 10 mM. Then, the HGC-27 and SGC-7901 cells were exposed to combinations of cisplatin and sodium butyrate for 24 h or 48 h according to the drug dosing scheme in Table 1. Figure 1C shows that sodium butyrate combined with cisplatin remarkably inhibited the growth of GC cells to a greater extent than cisplatin alone in a time- and concentration-dependent manner. We generated Fa-CI plots using CompuSyn software, which showed the synergistic effects of the combination of sodium butyrate and cisplatin in inhibiting the viability of GC cells (Figure 1D). Using the combination of sodium butyrate (0.5 mM) with cisplatin to treat GC cells caused the IC50 value of cisplatin and sodium butyrate to be significantly lower than that of cisplatin alone (Figure 1B).

**TABLE 1:** Specific ways of adding drugs.

Cisplatin (µg/mL)	Sodium butyrate (mM)	Combination (cisplatin + sodium butyrate)
1	1	1 + 1
2	2	2 + 2
4	4	4 + 4
8	8	8 + 8
16	16	16 + 16
32	32	32 + 32
64	64	64 + 64

### Sodium Butyrate Combined With Cisplatin Inhibited the Migration and Invasion of Gastric Cancer Cells

**FIGURE 2:**
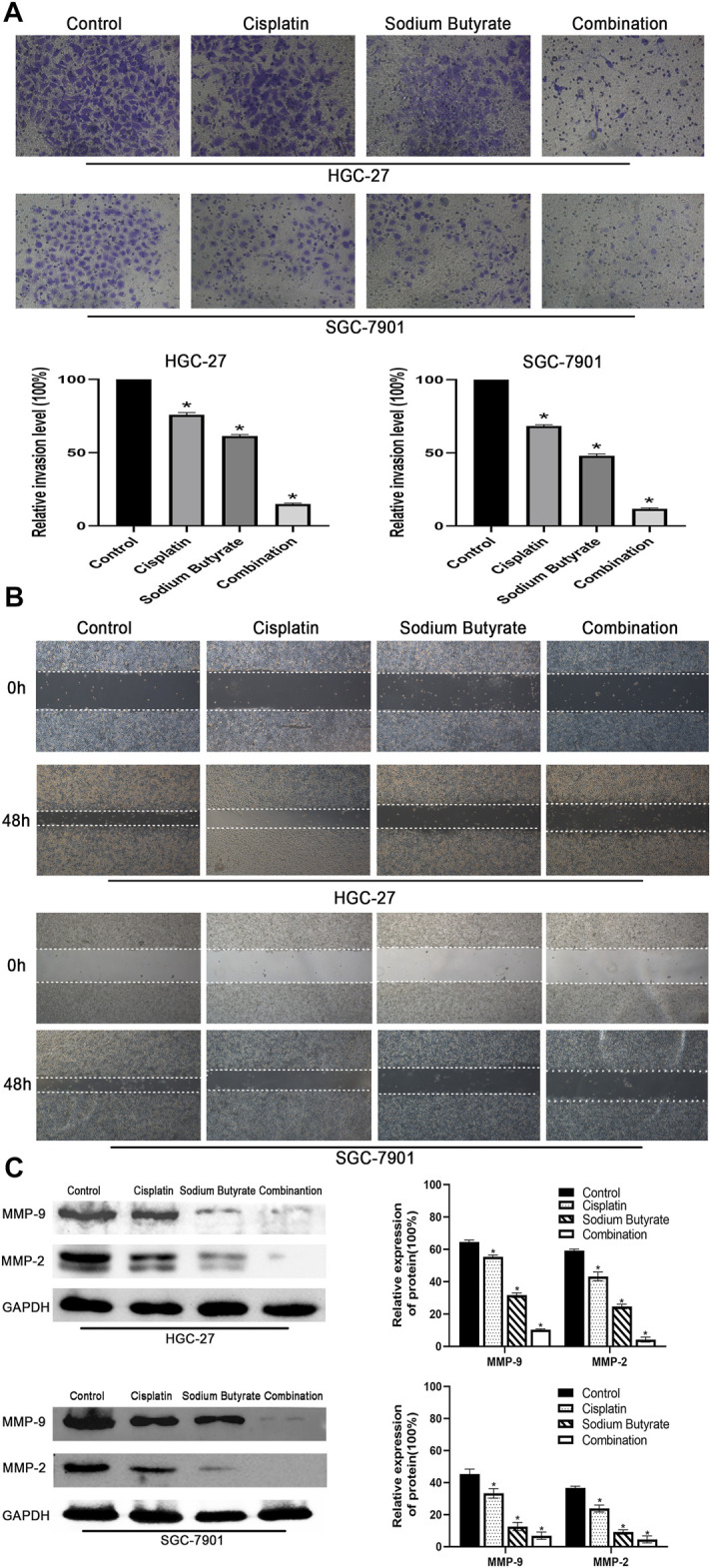
Effects of sodium butyrate and cisplatin on the invasion and migration of GC cells using the Transwell invasion assay and the wound-healing assay. **(A)** Original magnification: ×200. HGC-27 cells were treated with control, 4 µg/ml cisplatin, 10 mM soiudm butyrate, or 4 µg/ml cisplatin +10 mM sodium butyrate; SGC-7901 cells were treated with control, 4 µg/ml cisplatin, 5 mM sodium butyrate, or 4 µg/ml cisplatin +5 mM sodium butyrate. Quantitative analysis of the average invasive cell numbers in each group. *p < 0.05 vs the control group. **(B)** Cells were incubated with control, cisplatin, sodium butyrate, or cisplatin + sodium butyrate described in **(A)** above. **(C)** The levels of MMP-9 and MMP-2 were measured using western blotting and quantitative analysis of the proteins was performed. *p < 0.05 vs the control group. All the above data are the mean ± SD from an average of three experiments.

Wound-healing assays and Transwell invasion assays were used to measure the invasion and migration of GC cells. According to the results shown in Figures 2A,B, the cells in the combination treatment group had poorer invasion and migration abilities than the other groups. Meanwhile, western blotting was used to check the levels of MMP-2 and MMP-9 proteins, which showed that the combination group had the lowest levels of MMP-2 and MMP-9 proteins among the groups (Figure 2C).

### Sodium Butyrate Combined With Cisplatin Promoted Apoptosis in GC Cells

**FIGURE 3:**
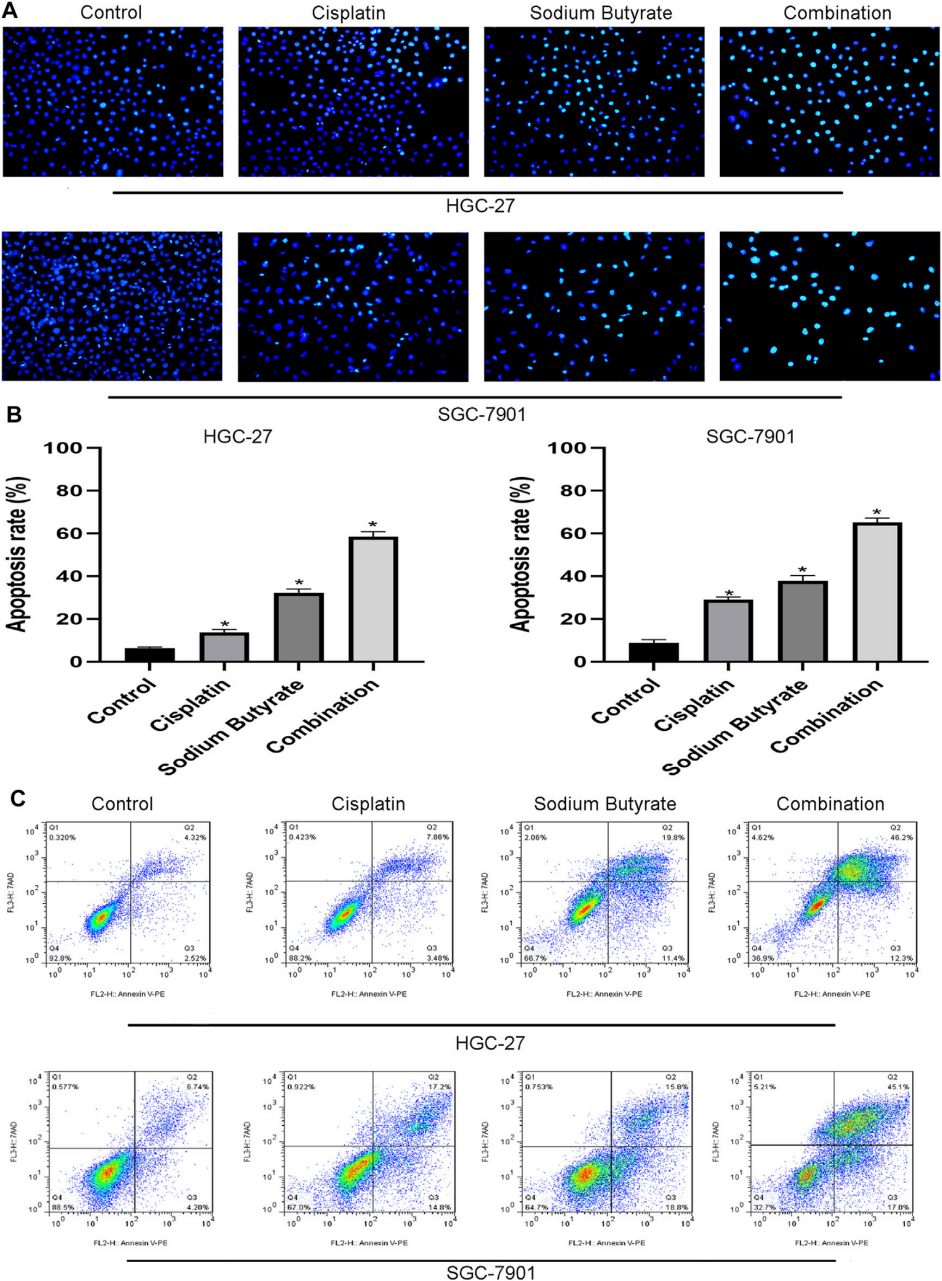
Sodium butyrate combined with cisplatin promotes apoptosis of GC cells. **(A)** Original magnification: ×200. HGC-27 cells were treated with control, 4 µg/ml cisplatin, 10 mM sodium butyrate, or 4 µg/ml cisplatin +10 mM sodium butyrate; SGC-7901 cells were treated with control, 4 µg/ml cisplatin, 5 mM sodium butyrate, or 4 µg/ml cisplatin +5 mM sodium butyrate. **(B)** Quantitative analysis of the apoptosis rate in each group. *p < 0.05 vs the control. **(C)** Quantitative flow cytometry measurements of apoptosis in GC cells. All the above data are the mean ± SD from an average of three experiments.

Hoechst 33,258 staining was used to evaluate the nuclei of GC cells exposed to sodium butyrate and/or cisplatin. The normal cell nuclei showed blue fluorescence, while the apoptotic cell nuclei showed bright blue fluorescence with fragmentation and chromatin condensation. The randomly selected fields of view showed that sodium butyrate combined with cisplatin promoted the apoptosis to a greater extent than that in the other groups of GC cells (Figures 3A,B). Annexin V-PE/7AAD staining further confirmed that sodium butyrate plus cisplatin promoted apoptosis to a greater extent than did the other treatments (Figure 3C).

### Sodium Butyrate Combined With Cisplatin Promotes Apoptosis of Gastric Cancer Cells via the Mitochondrial Apoptosis Pathway

**FIGURE 4:**
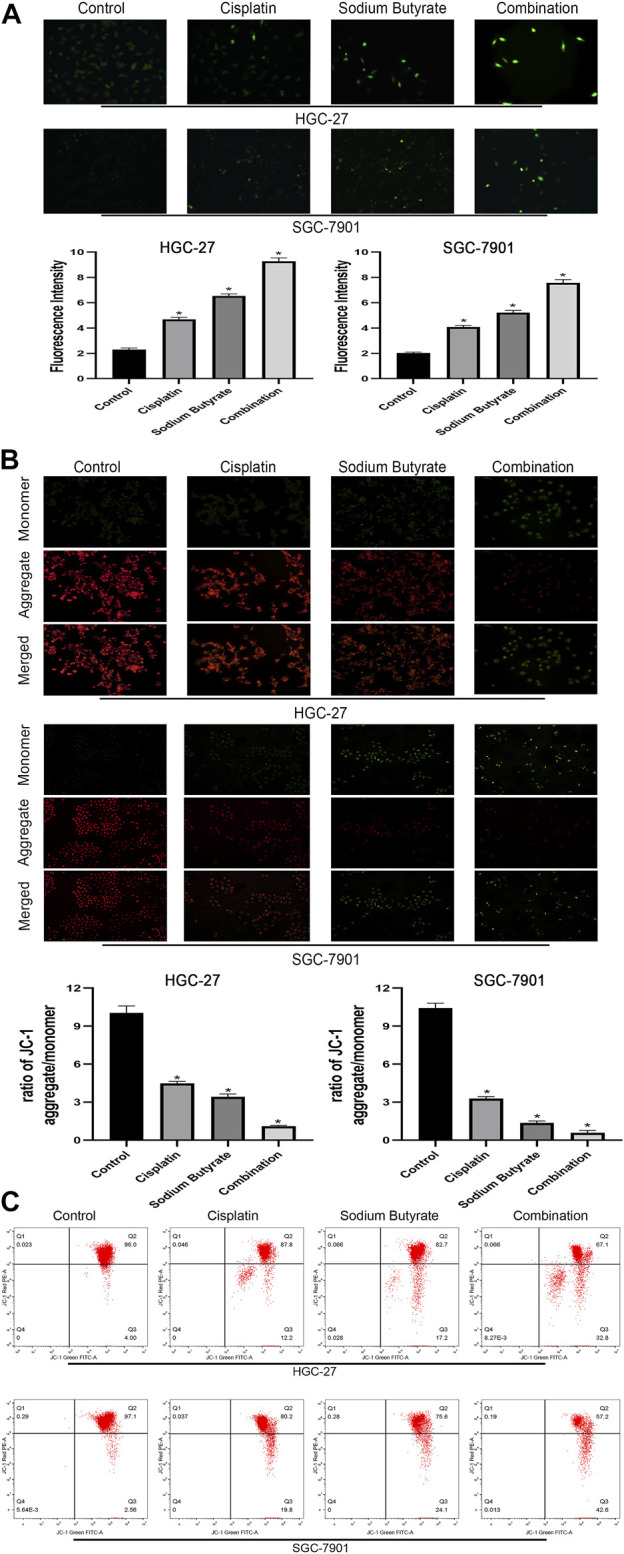
Sodium butyrate combined with cisplatin promotes the accumulation of intracellular ROS and the decreasing trend of the MMP. **(A)** Original magnification: ×200. HGC-27 cells were treated with control, 4 µg/ml cisplatin, 10 mM sodium butyrate, or 4 µg/ml cisplatin +10 mM sodium butyrate; SGC-7901 cells were treated with control, 4 µg/ml cisplatin, 5 mM sodium butyrate, or 4 µg/ml cisplatin +5 mM sodium butyrate. Quantitative analysis of ROS in each group. *p < 0.05 vs the control group. **((B)** and **(C))** Cells were incubated with control, cisplatin, sodium butyrate, or cisplatin + sodium butyrate described in **(A)** above. MMP was observed via JC-1 staining. Red inflorescence indicates healthy mitochondria; green inflorescence indicates collapsed mitochondrial potential. Quantitative analysis of the MMP in each group. *p < 0.05 vs the control group. All the above data are the mean ± SD from an average of three experiments.

To determine whether sodium butyrate combined with cisplatin facilitates the apoptosis of GC cells through the mitochondrial apoptosis pathway, changes in ROS levels and mitochondrial membrane potential (ΔΨm) levels were assessed. Figure 4A shows that the combination group accumulated more ROS (green fluorescence) than the other groups of GC cells. In Figures 4B,C, the fluorescence ratio of the JC-1 polymer of the combined drug group was the lowest among the four groups, indicating a decrease in the ΔΨm.

**FIGURE 5:**
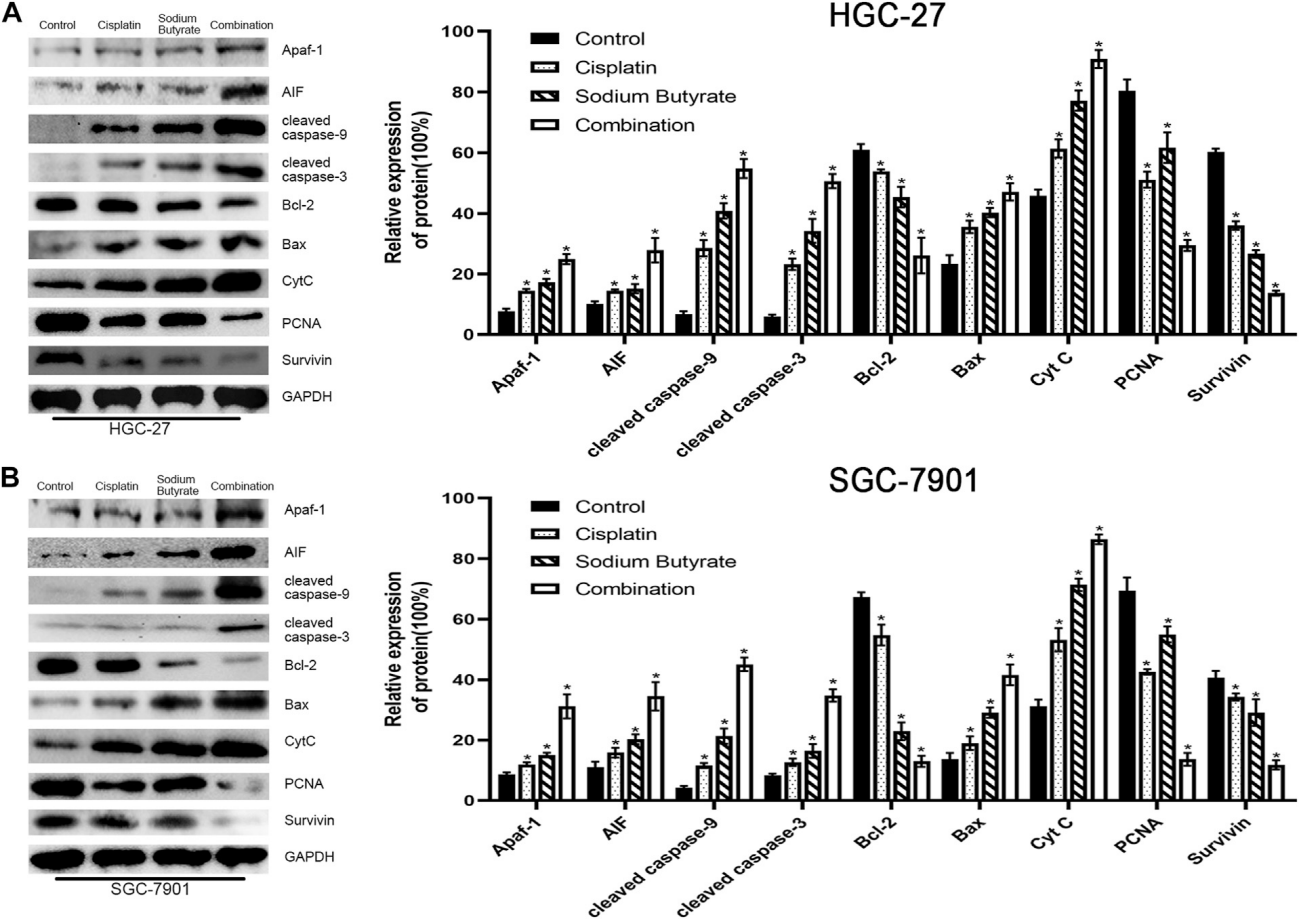
Sodium butyrate combined with cisplatin induces apoptosis through the mitochondrial pathway. **(A)** HGC-27 cells were treated with control, 4 µg/ml cisplatin, 10 mM sodium butyrate, or 4 µg/ml cisplatin +10 mM sodium butyrate, and western blotting was performed to detect the levels of related proteins. *p < 0.05 vs the control group. **(B)** SGC-7901 cells were treated with control, 4 µg/ml cisplatin, 5 mM sodium butyrate, or 4 µg/ml cisplatin +5 mM sodium butyrate, and western blotting was performed to detect the levels of related proteins. *p < 0.05 vs the control group. All the above data are the mean ± SD from an average of three experiments.

Western blotting was used to evaluate the levels of mitochondrial apoptosis pathway-related proteins to further validate the relationship between the combination drug treatment and apoptosis promotion via the mitochondrial apoptosis pathway. Western blotting showed that the levels of Apaf-1, Bax, AIF, cleaved-caspase 3, cleaved-caspase 9, and CytC in the combination group were increased, whereas the survivin, PCNA, and Bcl-2 levels were decreased, and the extent of the increase or decrease was higher than that observed using either agent alone and compared with the control (Figure 5).

### Pretreatment With NAC or BSO Inhibited or Enhanced Apoptosis in GC Cells Promoted by the Combination of Sodium Butyrate and Cisplatin

**FIGURE 6:**
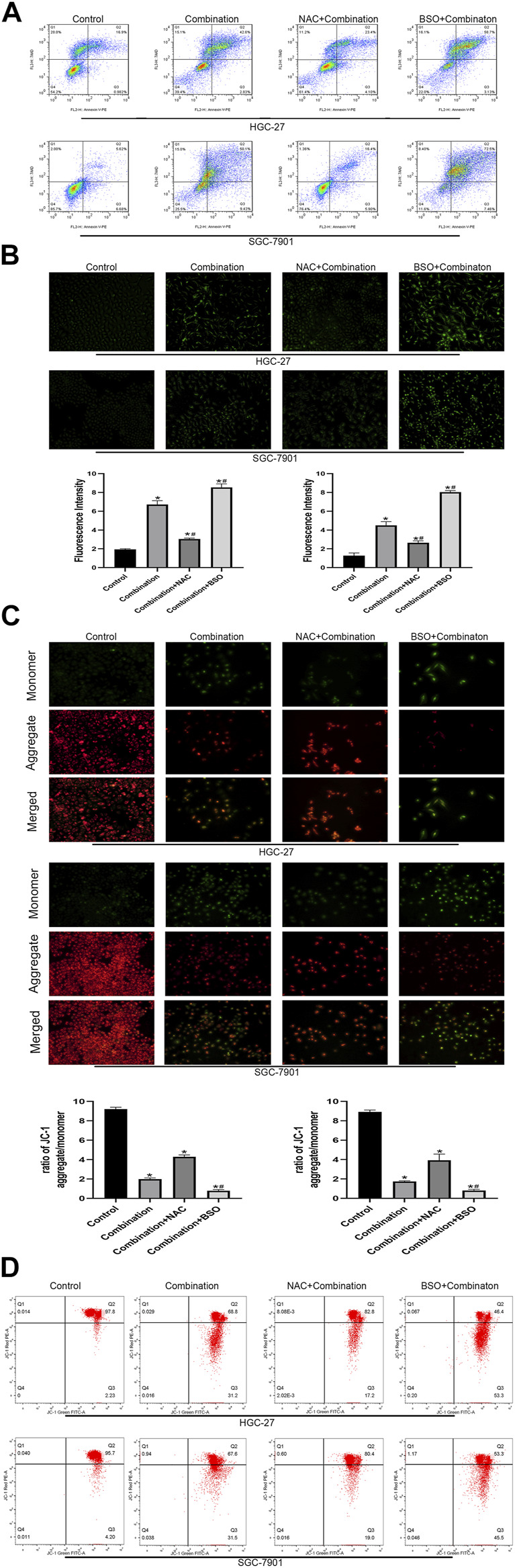
Pretreatment with NAC and BSO respectively influences the apoptosis of GC cells induced by soduim butyrate and cisplatin. **(A)** Quantitative flow cytometry measurements of apoptosis in HGC-27 cells (control, 4 µg/ml cisplatin +10 mM sodium butyrate, NAC pretreated + combination, or BSO pretreated + combination) and SGC-7901 cells (control, 4 µg/ml cisplatin +5 mM sodium butyrate, NAC pretreated + combination, or BSO pretreated + combination). **(B)** Original magnification: ×200. Cells were incubated with control, combination, NAC pretreatment + combination, or BSO pretreatment + combination described in **(A)** above. Quantitative analysis of ROS in each group. *p < 0.05 vs the control group; #*p* < 0.05 vs the combination group. **((C)** and **(D))** Cells were incubated with control, combination, NAC pretreatment + combination, or BSO pretreatment + combination described in **(A)** above. MMP was observed via JC-1 staining. Red inflorescence indicates healthy mitochondria; green inflorescence indicates collapsed mitochondrial potential. Quantitative analysis of MMP in each group. *p < 0.05 vs the control group; #*p* < 0.05 vs the combination group. All the above data are the mean ± SD from an average of three experiments.

To further clarify the molecular mechanisms associated with the increased apoptosis via the mitochondrial pathway, a glutathione (GSH) inhibitor (BSO) and promotor (NAC) were used to pretreat the GC cells for 2 h before treatment with the combined drugs. Annexin PE/7AAD staining showed that the number of apoptotic cells was decreased (NAC group) or enhanced (BSO group) (Figure 6A). The ROS levels in the combined treatment group were decreased (NAC group) or increased (BSO group) after 2 h of pretreatment (Figure 6B). NAC pretreatment reversed the decrease in MMP levels induced by the combined treatment, while BSO pretreatment promoted the decrease in MMP levels induced by the combined treatment (Figures 6C,D).

**FIGURE 7:**
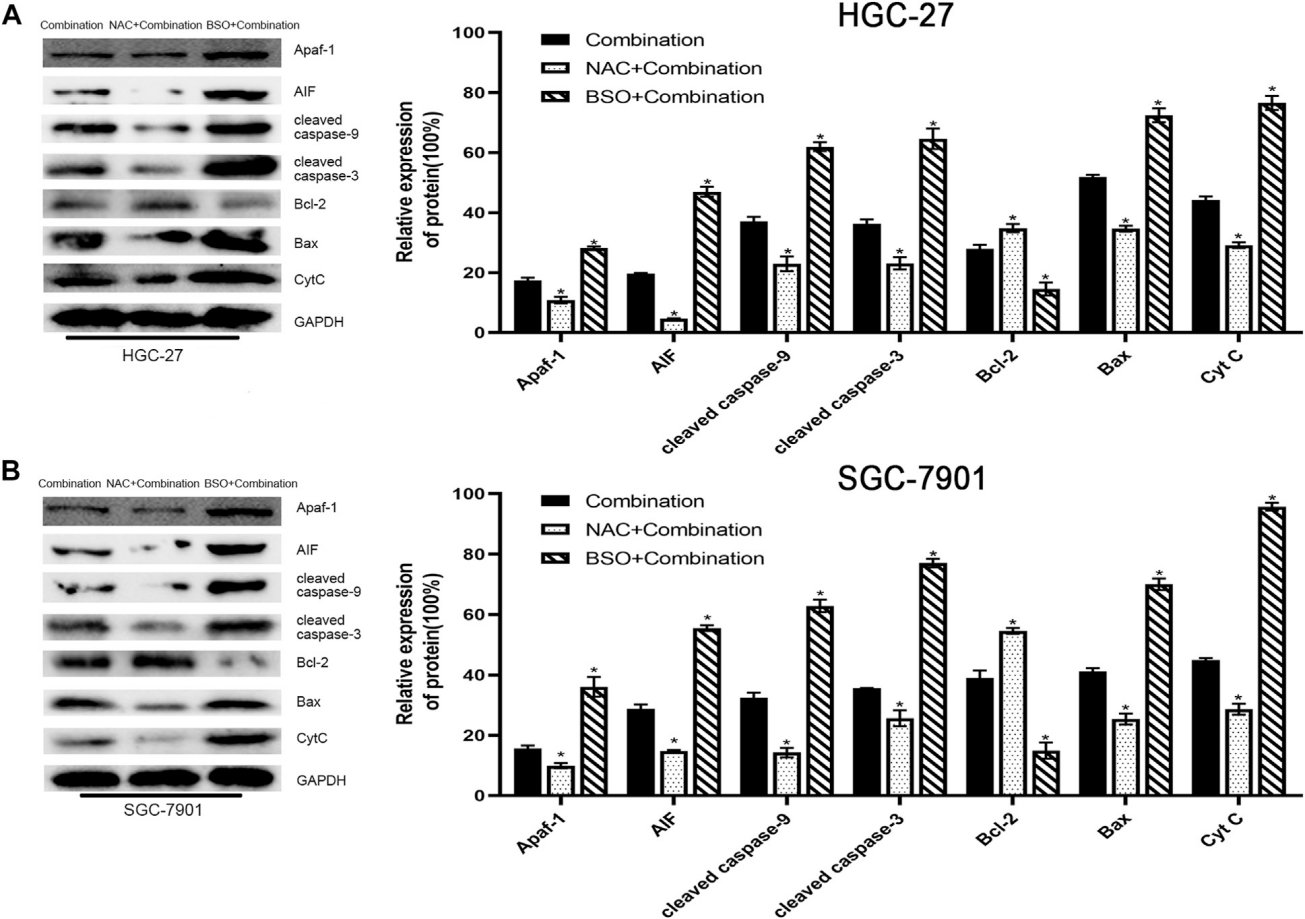
Pretreatment with NAC or BSO, respectively, decreased or increased the levels of mitochondrial apoptosis pathway-related proteins. **(A)** HGC-27 cells were treated with 4 µg/ml cisplatin +10 mM sodium butyrate, NAC pretreatment + combination, or BSO pretreatment + combination and western blotting was performed to detect the levels of related proteins. *p < 0.05 vs the combination group. **(B)** SGC-7901 cells were treated with 4 µg/ml cisplatin +5 mM sodium butyrate, NAC pretreatment + combination, or BSO pretreatment + combination and western blotting was performed to detect the levels of related proteins. *p < 0.05 vs the combination group. All the above data are the mean ± SD from an average of three experiments.

In addition, western blotting showed that NAC pretreatment decreased the levels of mitochondrial apoptosis pathway-related proteins, whereas BSO pretreatment increased their levels (Figure 7).

### Anti-Tumor Effects of Sodium Butyrate and Cisplatin on GC Cells *In Vivo*

**FIGURE 8:**
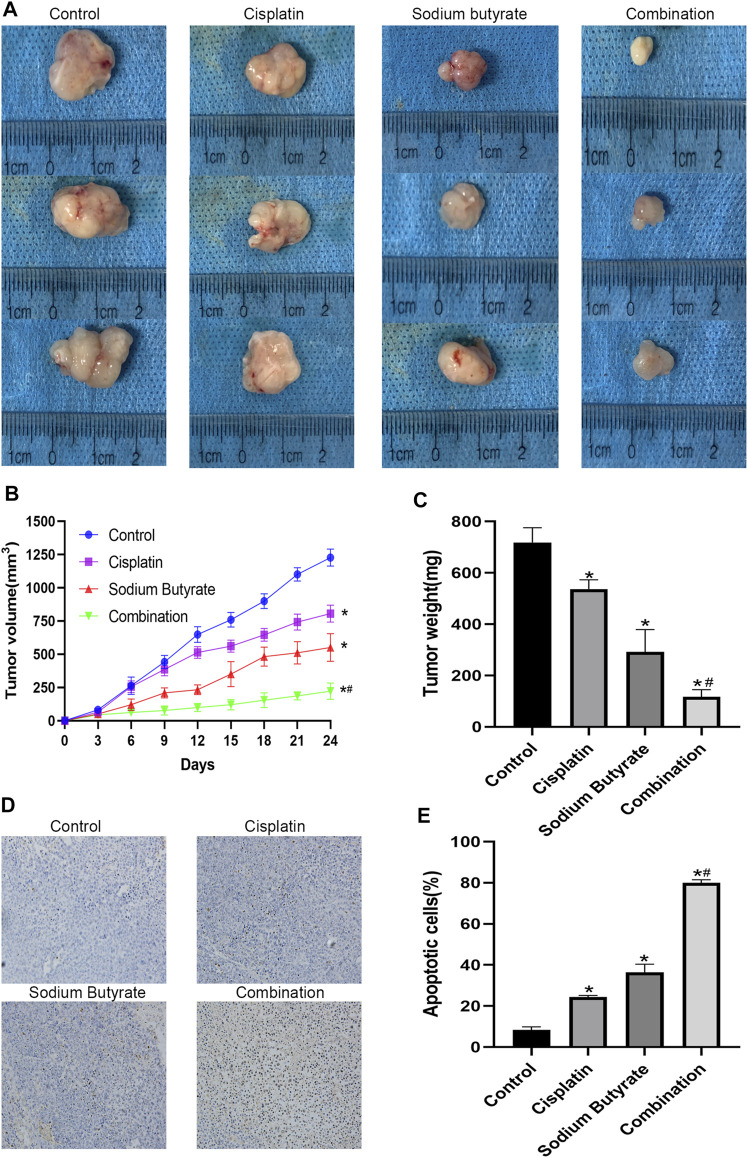
Anti-tumor effects of cisplatin and sodium butyrate *in vivo*. **(A)** Morphology of the subcutaneous implanted tumor. **(B)** Mean tumor volume at each time point. **(C)** The tumor weight obtained at the end of the experiment. **(D)** A TUNEL assay was performed to detect the apoptotic cells in the tumor tissue. **(E)** Quantitative analysis of the apoptosis rate in **(D)**. *p < 0.05 vs the control; *#*p* < 0.05 vs cisplatin alone. All the above data are the mean ± SD from an average of three experiments.

We carried out an *in vivo* study to explore the effects of cisplatin and/or sodium butyrate on the xenograft tumor growth. Compared with the control group, all the treatment groups showed inhibited growth of tumors *in vivo*, with significantly decreased tumor weight and volume; the best effect was achieved in the combination group (Figures 8A–C).

The TUNEL assay and hematoxylin and eosin (H&E) staining were performed on the tumor tissues isolated from the xenograft mice. In the three treatment groups, the TUNEL staining showed significant cell apoptosis in the tumors and the highest level of cell apoptosis was achieved in the combined treatment group (Figures 8D,E). Table 2 shows the serum BUN, AST, ALT, and Cr levels of blood samples, which assessed liver and kidney dysfunction; there was no visible difference among the four groups for any of these indices (*p* > 0.05).

**TABLE 2:** Effect of sodium butyrate combined with cisplatin or alone on hepatic and renal function.

Group	ALT (U/I)	AST (U/I)	Urea (µmol/l)	Cr (µmol/l)
Control	31.8 ± 2.40	143.3 ± 10.35	8.12 ± 0.44	13.00 ± 1.90
Cisplatin	34.2 ± 2.14	140.8 ± 7.63	8.63 ± 0.84	14.33 ± 1.37
Sodium butyrate	30.8 ± 2.79	146.0 ± 15.24	8.54 ± 0.68	13.67 ± 1.75
Combination	30.5 ± 2.35	142.2 ± 3.19	8.38 ± 0.61	14.17 ± 2.48

Data are presented as the mean ± standard deviation, with n = 6 mice/group. There were no differences in the ALT, AST, urea, and Cr levels among all groups (*p* > 0.05).

## Discussion

Anti-cancer drugs mainly promote tumor cell apoptosis to exert their effects. There are three main apoptotic pathways: the death receptor pathway, the mitochondrial pathway, and the endoplasmic reticulum stress pathway (Gupta et al., 2009; Lee et al., 2012; Iurlaro and Muñoz-Pinedo, 2016). The mitochondrial pathway, also known as the endogenous pathway, is an evolutionarily highly conserved form of cell death that plays an essential role in the development and homeostasis of multicellular organisms (Roca-Agujetas et al., 2019). The alteration of mitochondrial permeability plays a key role in the mitochondrial apoptotic pathway, which is achieved by the opening of the mitochondrial permeability transition pore (MPTP) (Sileikyte and Forte, 2019). MPTP opening is a central physiological event in maintaining the dynamic homeostasis of mitochondrial health (Barsukova et al., 2011). ROS mainly originate from the mitochondria and, in turn, the mitochondria are the targets of ROS (Venditti and Di Meo, 2020). Short-term, reversible MPTP opening releases a small amount of ROS, which is beneficial to the cell growth; however, continuous, irreversible opening of MPTP causes explosive release of ROS, leading to oxidative stress and damage to the mitochondria and the cells (Bolduc et al., 2019).

ROS are oxidants that promote apoptosis, playing the role of promoter and downstream carrier in the apoptosis process (Kirtonia et al., 2020). Reduced glutathione (GSH) is a key intracellular antioxidant that is important to maintain the proper redox state of sulfhydryl groups in proteins (Sun et al., 2018). The depletion of GSH plays an important role in the proliferation and apoptosis of tumor cells and induces the accumulation of ROS (Lv et al., 2019). As the accumulation of ROS reaches an irreversible point, the mitochondrial membrane oxidative stress is induced, allowing the MPTP to remain open, causing CytC and AIF to be released from the mitochondria into the cytoplasm (Izzo et al., 2016; Baechler et al., 2019). Subsequently, pro-apoptotic factors, such as CytC, interact with the caspase family factor, Apaf-1, and the Bcl-2 protein family are released into the cytoplasm to accelerate the apoptotic process (Burek et al., 2006). In contrast, survivin proteins, as antagonists of the caspase family, inhibit apoptosis (Martínez-García et al., 2018).

The increasing resistance to conventional chemotherapeutic agents, such as docetaxel and cisplatin, and the problem of cytotoxicity have led sodium butyrate, a metabolite produced by the intestinal flora, to be considered as a potential anticancer therapeutic agent (Gentilin et al., 2019; Chen et al., 2020). Intriguingly, a study confirmed that sodium butyrate modulated the gut microbiota beneficially and improved the host immune response in *in vivo* experiments (Ma et al., 2020). As a histone acetylation inhibitor, an increasing number of experiments have demonstrated that sodium butyrate can induce the apoptosis in a variety of cancer cells (Maruyama et al., 2012; Fialova et al., 2016; Mrkvicova et al., 2019; Xiao et al., 2020; Xi et al., 2021). Moreover, there is evidence that sodium butyrate works well in combination with other drugs in many cancers (Fialova et al., 2016; Taylor et al., 2019); however, whether sodium butyrate combined with cisplatin can increase the inhibition of GC cells has not been investigated. Almost all anti-cancer drugs exert their anti-cancer effects by activating the apoptotic pathway to overcome cancer non-surgically, and sodium butyrate is no exception. Sodium butyrate has been proven to induce apoptosis in carcinomas through the mitochondrial apoptotic pathway (Salimi et al., 2017; Qin et al., 2020); therefore, in the current study, we aimed to confirm that sodium butyrate combined with cisplatin promoted apoptosis in GC cells through a mitochondria-mediated signaling pathway.

The results of the present study showed that the combination group remarkably inhibited the growth of GC cells to a greater extent than cisplatin alone, dependent on the duration and concentration of the treatment. Subsequently, we confirmed the synergistic effect of the combination using CompuSyn software. In addition, a significant pro-apoptotic effect of the combined group toward gastric cancer cells was observed using a Hoechst assay and flow cytometry. To explore the pathway responsible for the observed apoptosis, we examined the intracellular ROS levels in the group of cells and found that the ROS levels in the combination group significantly exceeded those in the other three groups. Next, we detected the levels of the fluorescent dye JC-1 in GC cells to show the mitochondrial membrane potential of each group. We found that the accumulation of ROS led to changes in the mitochondrial membrane potential of GC cells, which induced apoptosis. Subsequently, we pretreated the combination group of GC cells with BSO and NAC and found significant differences in the apoptosis rate, ROS levels, and mitochondrial membrane potential levels in GC cells compared with those in the control group (Figure 6), which confirmed our hypothesis that the combination treatment induced the apoptosis of GC cells via the mitochondrial apoptosis pathway.

To further demonstrate the role of the mitochondrial pathway in promoting apoptosis in GC cells after the combined drug treatment, we examined the expression levels of relevant proteins in the mitochondrial pathway using western blotting. The results showed that sodium butyrate combined with cisplatin remarkably increased the levels of the pro-apoptotic proteins (Apaf-1, AIF, BAX, CytC, cleaved caspase-3, and cleaved caspase-9) and remarkably decreased the levels of anti-apoptotic proteins (BCL2, PCNA, and survivin). In addition, the increases in mitochondrial apoptosis pathway-related proteins as mentioned above were reversed after pretreatment with NAC or enhanced after pretreatment with BSO (Figure 7). Finally, the anti-cancer effects of the combined drugs on GC cells were tested *in vivo*, and the results showed that the combined drug treatment inhibited the proliferation of GC cells and significantly increased the number of apoptotic cells in tumors (Figure 8).

In conclusion, our results supported the hypothesis that sodium butyrate combined with cisplatin enhances apoptosis in GC cells through the mitochondrial apoptosis pathway *in vitro* and *in vivo*. Thus, sodium butyrate, a histone acetylation inhibitor produced by intestinal flora fermentation, combined with cisplatin could represent a therapeutic option to treat GC.

## Data Availability

The raw data supporting the conclusion of this article will be made available by the authors, without undue reservation.
